# Supplementary material for: Content-rich biological network constructed by mining PubMed abstracts
Source: BMC Bioinformatics. 2004 Oct 8;5:147. doi: 10.1186/1471-2105-5-147 (PMC528731; doi:10.1186/1471-2105-5-147)
Supplement: Additional File 5 — The original Chilibot query results of the term "long-term potentiation (LTP)" and 22 other terms, limiting the latest references analyzed to the years 1990, 1995, 2000, and 2004. [file 1471-2105-5-147-S5.bz2 › chilibotAdditionalFile5/ltp2004/html/AMPA_ACTININ.html]

 


 **AMPA** and **ACTININ** 
  
Found 8 abstracts in PubMed,  **8 abstracts were retrieved and analyzed**.  


---

 Search Google  |
 PDF files only 
|  EDU domain only 

---

**Interactive relationship** (e.g. stimulation, inhibition, etc)

- Immunoprecipitation of membrane protein extracts showed that alpha  **actinin**  2 is present in heteromeric complexes of NMDA subunits, but is not associated with  **AMPA**  receptors in the striatum.  Ref: 10925145 Brain Res Mol Brain Res, 2000
- Viral nucleoproteins, overexpressed using the Semliki Forest virus system, were localized to the dendritic spines as shown by double immunolabeling with  **actinin** , but did not by themselves cause neuronal death or changes in synaptic transmission as measured by  **AMPA**  mediated excitatory postsynaptic currents.  Ref: 11489350 Brain Res Bull, 2001
